# Neutrophils and neutrophil serine proteases are increased in the spleens of estrogen-treated C57BL/6 mice and several strains of spontaneous lupus-prone mice

**DOI:** 10.1371/journal.pone.0172105

**Published:** 2017-02-13

**Authors:** Rujuan Dai, Catharine Cowan, Bettina Heid, Deena Khan, Zhihong Liang, Christine T. N. Pham, S. Ansar Ahmed

**Affiliations:** 1 Infectious Disease Research Facility (IDRF), Department of Biomedical Sciences and Pathobiology, Virginia-Maryland College of Veterinary Medicine (VMCVM), Virginia Tech, Blacksburg, Virginia, United States of America; 2 Department of Medicine, Division of Rheumatology, Washington University School of Medicine, St. Louis, Missouri, United States of America; Universitat Bern, SWITZERLAND

## Abstract

Estrogen, a natural immunomodulator, regulates the development and function of diverse immune cell types. There is now renewed attention on neutrophils and neutrophil serine proteases (NSPs) such as neutrophil elastase (NE), proteinase 3 (PR3), and cathepsin G (CG) in inflammation and autoimmunity. In this study, we found that although estrogen treatment significantly reduced total splenocytes number, it markedly increased the splenic neutrophil absolute numbers in estrogen-treated C57BL/6 (B6) mice when compared to placebo controls. Concomitantly, the levels of NSPs and myeloperoxidase (MPO) were highly upregulated in the splenocytes from estrogen-treated mice. Despite the critical role of NSPs in the regulation of non-infectious inflammation, by employing NE^-/-^/PR3^-/-^/CG^-/-^ triple knock out mice, we demonstrated that the absence of NSPs affected neither estrogen’s ability to increase splenic neutrophils nor the induction of inflammatory mediators (IFNγ, IL-1β, IL-6, TNFα, MCP-1, and NO) from *ex vivo* activated splenocytes. Depletion of neutrophils *in vitro* in splenocytes with anti-Ly6G antibody also had no obvious effect on NSP expression or LPS-induced IFNγ and MCP-1. These data suggest that estrogen augments NSPs, which appears to be independent of enhancing *ex vivo* inflammatory responses. Since estrogen has been implicated in regulating several experimental autoimmune diseases, we extended our observations in estrogen-treated B6 mice to spontaneous autoimmune-prone female MRL-*lpr*, B6-*lpr* and NZB/W_F1_ mice. There was a remarkable commonality with regards to the increase of neutrophils and concomitant increase of NSPs and MPO in the splenic cells of different strains of autoimmune-prone mice and estrogen-treated B6 mice. Collectively, since NSPs and neutrophils are involved in diverse pro-inflammatory activities, these data suggest a potential pathologic implication of increased neutrophils and NSPs that merits further investigation.

## Introduction

Estrogen has been shown to regulate the immune system of both normal and autoimmune individuals either via activation of estrogen receptor α (ERα) and/or ERβ or through ER-independent mechanisms [1–5]. It has been reported that *in vivo* estrogen exposure promotes the production of inflammatory cytokines such as interferon-gamma (IFNγ), Interleukin (IL)-6, IL-1β, chemokines such as monocyte chemoattractant protein (MCP)-1 and MCP-5), and inflammatory molecules such as nitric oxide (NO) in Concanavalin A (Con A) or lipopolysaccharide (LPS)-activated mouse splenic lymphoid cells and/or peritoneal macrophages [6–9]. Further, estrogen is capable of promoting B cell survival and activation or breakdown of B cell tolerance to induce lupus-related serology and pathology in non-autoimmune mice [10–13]. Together, these data demonstrate a pivotal role of estrogen in the regulation of T and B lymphocyte-mediated inflammation and autoimmune responses. While the regulatory role of estrogen on T and B lymphocytes is well documented, its effects on neutrophils remains largely unknown.

Neutrophils, a major leucocyte subset of innate immune cells, are the first line of cellular defense against invading pathogens. Neutrophils counter invading pathogens via a variety of mechanisms that include phagocytosis, respiratory burst, and recently identified NET*osis* [14, 15]. Neutrophil derived serine proteases (NSPs) including neutrophil elastase (NE), proteinase 3 (PR3), and cathepsin G (CG) are essential for neutrophils scavenging of infectious agents. In addition, NSPs play important roles in the regulation of non-infectious inflammatory responses via proteolytic processing of cytokines, chemokines, and signaling molecules such as NF-κB and p21 [14, 16, 17]. Furthermore, NSPs can regulate inflammation via activation of cell surface receptors such as integrins, protease-activated receptors (PARs), and toll-like receptors (TLRs) [14, 16–18]. In addition to their primary role in innate immune responses and inflammation, neutrophils are also critically involved in the adaptive immune response by attracting T cells to sites of inflammation and/or by priming and engaging T cell activation [19, 20].

Sex differences in neutrophil counts have long been observed in men and women. Women usually have higher neutrophil numbers than men [21], which could, in part, contribute to stronger immune responses in women compared to men. The potential effect of estrogen on neutrophils is suggested by the observation that the neutrophil counts in women fluctuate during the menstrual cycle and that increased neutrophil percentages are associated with higher serum estradiol [22–24]. To date, there is limited data with regard to estrogen effects on neutrophils and neutrophils-mediated onsite inflammatory responses. Previous studies in 1980s’ have documented that estrogen impaired hematopoiesis with decreased bone marrow cellularity, causing lymphopenia and neutropenia in estrogen-treated mice [25, 26]. A later study however has shown that estrogen and its metabolites were able to stimulate granulocytic differentiation in myoblasts and induced neutrophilia in mice [27]. Estrogen treatment was able to reduce the vascular injury response via inhibiting inflammatory mediator production and then attenuating neutrophil infiltration to injured arteries [28]. However, in a different murine influenza infection model, estrogen treatment enhanced pulmonary recruitment of neutrophils to potentiate virus-specific CD8^+^ T cells response for virus clearance [29]. This suggests that the effect of estrogen on neutrophils depends on the experimental context and tissue type.

In this study, we investigated the estrogen effect on neutrophils in bone marrow, blood and splenic lymphoid tissues to enable a better understanding of the potential broad tissue effects of estrogen on neutrophils. Our study clearly shows that estrogen upregulates neutrophils in the above lymphoid organs and promotes NSP expression in whole splenocytes. Abnormal expression and function of NSPs has been implicated in the pathogenesis of many chronic autoimmune inflammatory diseases including SLE [14, 30]. Nevertheless, depletion of NSPs *in vivo* in mice and depletion of neutrophils *in vitro* in splenocytes had no obvious effect on LPS induced inflammatory responses in splenocytes of estrogen-treated mice. This suggests that increased neutrophils and NSPs are not directly involved in estrogen-mediated promotion of inflammatory responses. Since estrogen has been reported to induce lupus-related autoimmune inflammatory parameters[10–13], we therefore investigated whether there are also changes of neutrophils and NSPs in the spleen of three different genetically lupus-prone murine models (MRL-*lpr*, B6-*lpr*, and NZB/W_F1_), which manifest varied forms of lupus and other systemic autoimmune diseases. The finding of a similar augmentation of neutrophils and NSPs in the spleens of different spontaneous murine lupus models and estrogen-treated wild-type B6 mice suggests a potential significance of estrogen upregulation of neutrophils and NSPs in autoinflammation.

## Materials and methods

### Ethics statement and mice

All animal experimental procedures and housing have been approved by the Institutional Animal Care and Use Committee (IACUC) of Virginia Tech (Protocol ID# 12-131-CVM). The experimental mice were euthanized by cervical dislocation in strict accordance with approved IACUC protocol and regulations. To minimize suffering and to ensure a successful euthanasia of mice within seconds, cervical dislocation was carried out only by well-trained and approved research staff. All mice were housed in our AAALAC accredited animal facility at the Virginia-Maryland College of Veterinary Medicine (VMCVM), Virginia Tech. Mice were fed with a commercial 7013 NIH-31 Modified 6% Mouse/Rat Sterilizable Diet (Harlan Laboratory, Madison, WI, USA) and given water *ad libitum*.

Three to four week-old male C57BL/6 (B6) mice were purchased from the Charles River Laboratories, USA. The NE^-/-^/PR3^-/-^/CG^-/-^ triple knock out (NECGPR3 deficient[31], referred as NSP^-/-^ in the text) breeders on B6 background were kindly provided by Dr. Christine T.N. Pham from the Washington University Medical School and bred in our animal facility. Genetically autoimmune-prone female MRL/MpJ-*Fas*^lpr^/J (MRL-*lpr*, stock No: 006825), B6.MRL-Fas^lpr^/J (B6-lpr, stock No: 000482), NZBWF1/J (NZB/W_F1_, stock No: 100008), and their respective control female MRL/MpJ (MRL, stock No: 000486), B6, and NZW/LacJ (NZW, stock No: 001058) mice were purchased from The Jackson Laboratory, ME, USA. These three strains of mice spontaneously develop different manifestations of lupus and other forms of systemic autoimmune diseases. The female MRL-*lpr*, B6-*lpr*, and their control female MRL and B6 mice were euthanized at 3–4 months old; the female NZB/W_F1_ and its control female NZW mice were euthanized at 7–9 months old for the experimental analysis.

For estrogen implant treatment, the 4–5 week-old male B6 and NSP^-/-^ mice were orchidectomized and implanted with 17-β estradiol (Sigma-Aldrich, Saint Louis, MO, USA) or empty (placebo control) silastic implants following our standardized lab protocol [8, 32–34]. After seven to eight weeks of treatment, the mice were euthanized to isolate splenocytes and/or bone marrow cells for experimental analysis. The serum estrogen levels in estrogen-implanted mice generated by this standard lab practice have been reported previously (ranged from 140 to 270 pg/ml at 7 wks of treatment) [8, 33].

### Splenocyte preparation and culture

Mouse splenocytes were isolated using well-established lab procedures that have been described in detail before [8, 32–34]. The splenocytes were resuspended at 5 × 10^6^ cells/ml in phenol red free RPMI-1640 medium (Mediatech Inc, Manassas, VA, USA) supplemented with 10% charcoal-stripped fetal bovine serum (Atlanta Biologicals, Flowery Branch, GA, USA), 2 mM L-glutamine (HyClone Labs Inc, Logan, UT, USA), 100 IU/ml penicillin and 100 μg/ml streptomycin (HyClone), and 1% non-essential amino acids (HyClone). The inadvertent potential estrogenic effect of culture media was carefully avoided by the use of phenol red free RPMI and charcoal-stripped fetal bovine serum.

To activate splenocytes, 2.5 x10^6^ cells were seeded in 24-well plates and stimulated with 500 pg/ml of LPS (Sigma-Aldrich) as designated in the ensuing figures and figure legends. The cell pellets were collected for Western blot analysis and the culture supernatants were collected for analysis of cytokine and chemokine levels by the ELISA assay.

### Western blotting

The whole cell extracts were prepared by lysing the cell pellet with CelLyticM Cell Lysis Reagent (Sigma-Aldrich). Western blotting was performed per our previously published protocol to analyze the protein expression in the whole cell extracts [33, 35]. The anti-NE (N18, sc-9518), anti-PR3 (p20, sc-19748), and anti-iNOS (M19, sc-650) antibodies were purchased from Santa Cruz Biotechnology Inc., Paso Robles, CA, USA. The anti-CC/DPPI (AF1034-SP) and anti-MPO (AF3667-SP) antibodies were purchased from Novus Biologicals, Littleton, CO, USA. The protein loading control antibody, anti-β-actin antibody (ab8227), was obtained from Abcam Inc., Cambridge, MA, USA.

### Protease and elastase activity assay

The EnZCheck protease Assay (E6638) kit and EnZCheck elastase assay (E12056) kit (Invitrogen, Grand Island, NY, USA) were used to measure the protease and elastase activities, respectively in whole splenocytes. Briefly, the freshly isolated splenocytes were lysed with CelLytic^™^M Cell Lysis Reagent (Sigma-Aldrich). All samples were adjusted with the lysis buffer to the same concentration, and equal amounts of protein from each sample were incubated with BODIPY^®^ FL dye conjugated substrates (casein for protease assay and DQ^™^ elastin for elastase assay) in 1X digestion buffer for 24 hrs. The fluorescence signal that reflects protease activity was measured by using a Spectra Max Gemini XPS microplate reader (Molecular Device, Sunnyvale, CA, USA) with excitation 485nm/emission 538nm. Since DQ^™^ elastin can also be digested by proteases other than elastase, a selective inhibitor of elastase N-methoxysuccinyl-Ala-Ala-Pro-Val-chloromethylketone (25μM-100 μM) was added to the separate parallel assay reaction following manufacturer’s instruction to determine the specificity of elastase activity.

### Assay of serine protease activity in living splenocytes

The Fluorescent-Labeled Inhibitors of Serine Protease (FLISP)^™^ detection kits (ImmunoChemistry Technologies LLC, Bloomington, MN, USA) were utilized to measure intracellular serine protease activity in the living splenocytes. The Carboxyfluorescein (FAM)-Spacer-Phenylalanine (Phe)-Chloromethyl Ketone (CMK) FLISP assay kit (FSFCK) and FAM- Spacer-Leucine (Leu)-CMK FLISP assay kit (FSLCK) can detect chymotrypsin-like serine proteases that favor targeting amino acid phenylalanine and leucine, respectively. Briefly, 200 μl of splenocytes at 5x10^6^/ml were plated in U bottom 96 well culture plates and incubated with 7.5μM of FSFCK or FSLCK reagent at 37°C, 5% CO_2_ incubator for 1.5 to 2 hrs. Then, the cells were washed three times with wash buffer, resuspended with PBS buffer containing 0.5% BSA, and analyzed by Flow Cytometer to determine the FAM signal intensity, which reflects the serine protease activity.

### Depletion of neutrophils *in vitro* from splenocytes

The EasySep^™^ Mouse Streptavidin RapidSpheres^™^ Isolation Kit (STEMCELL Technologies Inc., Vancouver, BC, Canada) was used to deplete splenic neutrophils *in vitro*. Briefly, the splenocytes were collected and suspended in MACS buffer (PBS containing 0.5% BSA and 2 mM EDTA) at 1x10^8^/ml. Normal Rat serum (50 μl/ml) was added to the cells before the addition of specific antibody. To deplete neutrophils, biotin conjugated anti-mouse Ly6G or anti-mouse Gr-1 antibody (Biolegend, San Diego, CA, USA) was added into a 5 ml polystyrene round-bottom tube containing 1.5x10^7^ splenocytes (150 μl) at a concentration of 2 μg/ml and then incubated at RT for 10 minutes, and then incubation with streptavidin beads (25 μl/ml) at RT for 2.5 minutes. After adding 1.5 ml MACS buffer, the tube (without cap) was placed into magnet immediately, incubated at RT for 2.5 minutes. Subsequently, the neutrophil-depleted splenocytes were collected in a new collection tube, pelleted, and resuspended in complete RPMI medium at 5x10^6^/ml for cell culture. Aliquot of splenocytes that was incubated with streptavidin beads only was served as control (No-Ab control).

### Real-time RT-PCR

Total RNA was isolated from freshly prepared splenocytes and purified splenic cells subsets using RNAeasy mini kit (Qiagen, Valencia, CA, USA) or mirVana miRNA isolation kits (Ambion). Any contaminated residual DNA was removed either by performing on-column DNase digestion during RNA isolation (RNAeasy mini kit) or by using RQ1 RNase-free DNAase (Promega, Madison, WI, USA) after RNA is extracted (mirVana miRNA isolation kit) per the manufacturer’s instruction. The iScript one-step RT-PCR with SYBR green kit (Bio-Rad, Hercules, CA, USA) was used for quantifying gene expression. Quantitect 10 × PCR primer mixes for mouse NE, PR3, CG, myeloperoxidase (MPO), and β-actin were purchased from Qiagen. The relative NSP mRNA expression levels were normalized to β-actin levels and calculated using the 2^−ΔΔCt^ (Livak) method.

### Detection of inflammatory molecules in culture supernatant

As reported previously [33–35], the level of the NO indicator, nitrite in cell culture supernatant was measured by the Griess assay. The levels of Th1 cytokines IFNγ, IL-1β, IL-6, TNFα, and Th2 cytokine IL-10 were measured by Ciraplex multiplex Chemiluminescent Assay kit (Aushon Biosystem Inc., Billerica, MA, USA). The chemokine MCP-1 level was measured with mouse MCP-1 ELISA MAX^™^ Deluxe kit (Biolegend Inc., San Diego, CA, USA).

### Statistical analysis

All values were reported as mean ± SEM. Two tailed, unpaired *t* tests were performed to assess statistical significance of mRNA expression levels, protease activities between two biological groups (placebo *vs* estrogen; MRL *vs* MRL-*lpr*; B6 *vs* B6-*lpr*; NZW *vs* NZB/W_F1_). Paired *t* tests were performed to assess the statistical significance between control and specific treated samples (without inhibitor *vs* with inhibitor; No-Ab control *vs* anti-Ly6G or anti-Gr-1).

## Results

### Increased protease and elastase activity in splenocytes from estrogen-treated B6 mice

**Fig 1:**
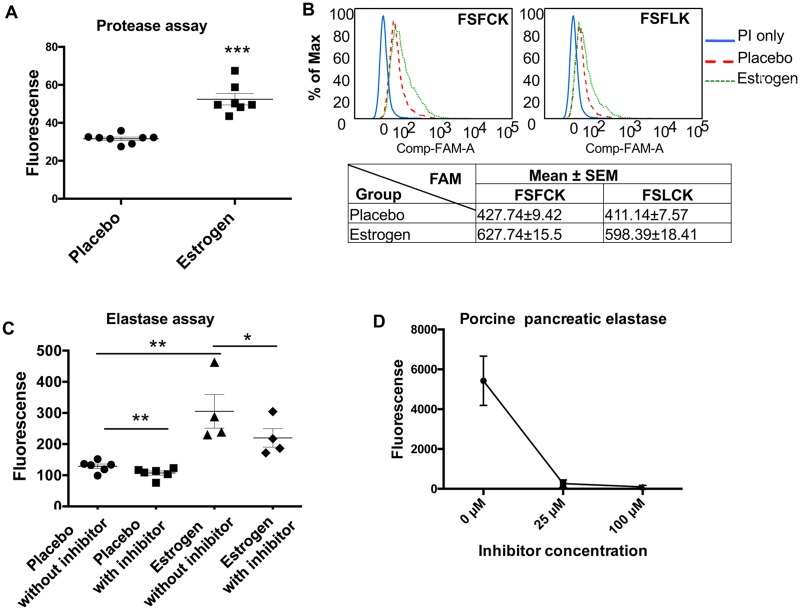
The protease and elastase activities are increased in splenocytes from estrogen-treated mice when compared to placebo controls. (A) The total protease activity in freshly isolated splenocytes from placebo- and estrogen-treated B6 mice was determined by EnzChek Protease Assay kit. The graph shows the mean ± SEM (n≥7). (B) The chymotrypsin-like serine protease activity in whole living splenocytes was detected with FLISP^™^ serine protease detection kit. Freshly isolated splenocytes were stained with FSFCK (upper left panel) or FSLCK (upper right panel) FLISP reagent, which detected chymotrypsin-like serine proteases favoring phenylalanine and leucine, respectively. The FAM signal intensity in living splenocytes (gated on propidium iodide (PI) negative cells) was analyzed by flow cytometry. The representative flow scatter plots are shown. The table in the lower panel shows the FAM value (mean ± SEM) summarized from the Flow data (n≥6). (C) The elastase activity in freshly isolated splenocytes from placebo- and estrogen-treated B6 mice was determined by EnZCheck elastase assay kit with or without the addition of inhibitor in the reaction. Mean activity ± SEM (n≥4) is shown. (D) The graph demonstrates that the activity of purified porcine pancreatic elastase was completely blocked by a selective inhibitor, N-methoxysuccinyl-Ala-Ala-Pro-Val-chloromethylketone at 100 μM concentration. Either unpaired student *t* test (placebo *vs* estrogen) or paired student *t* test (without inhibitor *vs* with inhibitor) were performed; *, *p*<0.05, ** *p*<0.01, and ***, *p* <0.001.

We have previously reported a serine protease mediated truncation of STAT-1 and NF-κBp65 proteins in the nuclear extracts of splenocytes from estrogen-treated B6 mice [35], which suggested a potential regulatory effect of estrogen on serine proteases. We therefore measured the total protease activity in whole cell lysates of freshly isolated splenocytes from placebo- and estrogen-treated mice. As expected, the protease activity was significantly increased in splenocyte lysates from estrogen-treated B6 mice compared to placebo controls (Fig 1A). By using FLISP Serine Protease Detection Assay, we further demonstrated that there was a significantly higher chymotrypsin-like serine protease activity in living splenocytes from estrogen-treated mice when compared to placebo controls (Fig 1B). We then measured the specific elastase activity in splenocytes using a commercially available EnZCheck elastase assay kit. As shown in Fig 1C, the protease activity was significantly increased in splenocytes from estrogen-treated mice (without inhibitor: placebo *vs* estrogen). The addition of a selective elastase inhibitor, N-methoxysuccinyl-Ala-Ala-Pro-Val-chloromethylketone (100 μM) significantly reduced the protease activity (estrogen: without inhibitor *vs* with inhibitor), suggesting a major contribution of elastase to the increased protease activity observed in splenocytes from estrogen-treated mice (Fig 1C). While the selective elastase inhibitor at 100 μM completely blocked the activity of the positive control (purified porcine pancreatic elastase, Fig 1D), it only partially inhibited the protease activity in splenocytes from estrogen-treated mice (Fig 1C). This suggests that in addition to elastase, estrogen also upregulated other types of proteases/serine proteases.

### Enhanced neutrophil serine protease expression in splenocytes from estrogen-treated B6 mice

**Fig 2:**
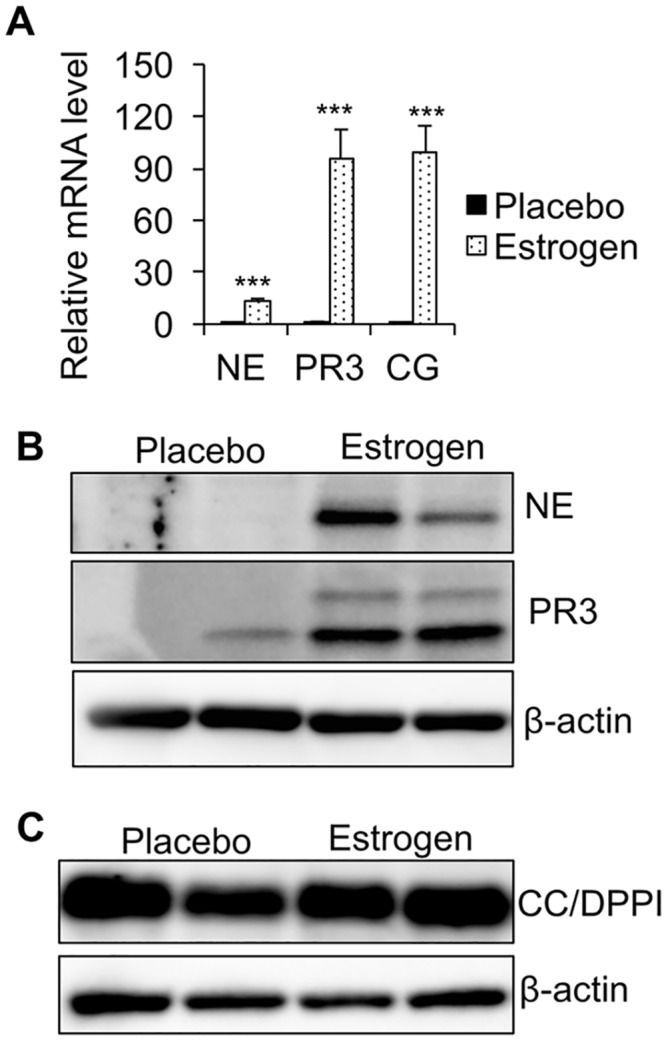
NSP expression levels are upregulated in the splenocytes from estrogen-treated B6 mice when compared to placebo controls. (A) Real-time RT-PCR analysis of the relative mRNA expression levels of NE, PR3, and CG in the splenocytes from placebo- and estrogen-treated B6 mice. The graph represents the means ± SEMs (n ≥ 4). Student *t* tests (placebo *vs* estrogen) were preformed; *, *p* < 0.05; **, *p* < 0.01; and ***, *p* < 0.001. (B and C) Western blot analysis of NE and PR3 (B), CC/DPPI (C) protein expression levels in the whole splenocyte extracts from placebo- and estrogen-treated mice. β-actin was probed as a protein loading control.

While there are a variety of serine proteases produced in different immune cell types, NSPs are of particular interest because of their role in the regulation of non-infectious inflammation[17]. With the finding of increased elastase activity in estrogen-treated splenocytes, we next examined whether estrogen treatment affects the expression of NE or other NSPs in splenocytes. Real-time RT-PCR analysis indicated that mRNA expression levels of all three NSPs (NE, PR3, and CG) were substantially increased in the freshly isolated splenocytes from estrogen-treated mice when compared to placebo controls (Fig 2A). Estrogen treatment led to an over 10-fold increase in NE mRNA level and an over 80-fold increase in PR3 and CG mRNA levels in splenocytes. In accordance with increased mRNA levels, the protein expression of NSPs such as NE and PR3 were also significantly enhanced in splenocytes from estrogen-treated B6 mice when compared to placebo controls (Fig 2B). We were unable to analyze CG protein level in these samples because the CG antibody does not work well with our western blotting system. Cathepsin C (CC, also called Dipeptidyl peptidase I (DPPI)) is required for the full activation of neutrophil derived NE, PR3, and CG [36]. Western blotting revealed similar expression levels of CC/DPPI in the splenocytes of placebo and estrogen-treated mice (Fig 2C).

### *In vivo* estrogen exposure increases the percentage of neutrophils in spleen, blood, and bone marrow

**Fig 3:**
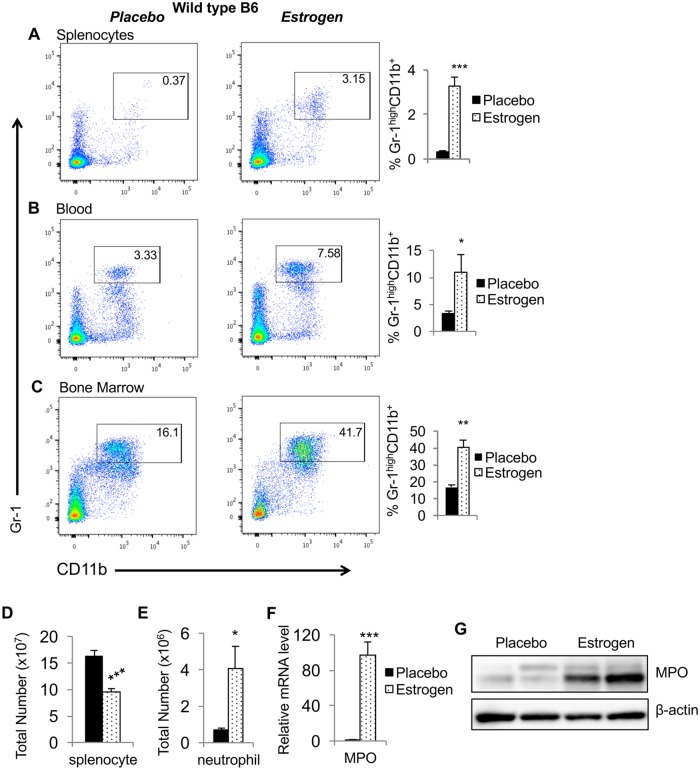
Estrogen treatment increases neutrophil percentages in wild type B6 mice. (A-C) Flow cytometry analysis. Red blood cell-depleted whole splenocytes (A), peripheral blood (B), and bone marrow (C) cells from placebo- and estrogen-treated mice were stained with neutrophil surface markers FITC conjugated anti-Gr1 and APC conjugated anti-CD11b antibodies. Shown are the representative FACS plots from at least three independent experiments accompanied by the summary graphs showing the percentages (means ± SEMs) of neutrophils in the spleen, blood, and bone marrow cells from placebo- and estrogen-treated mice (n≥4). (D) The total splenocyte count in placebo- and estrogen-treated mice. (E) The total CD11b^+^GR1^+^ splenic neutrophil count in placebo- and estrogen-treated mice. (F) Real-time RT-PCR analysis of the expression of MPO in splenocytes from placebo- and estrogen-treated mice. The graphs show means ± SEMs (n≥4). Unpaired student *t* tests (placebo *vs* estrogen) were performed. *, *p* < 0.05; **, *p* < 0.01; and ***, *p* < 0.001. (G) Western blot analysis of MPO protein expression levels in the splenocytes from placebo- and estrogen-treated mice. β-actin was probed as protein loading control.

We next determined whether estrogen alters the percentage of neutrophils in various lymphoid tissues. The flow cytometric analysis revealed that the percentage of mature neutrophils (Gr-1^high^CD11b^+)^ was significantly increased in splenocytes (Fig 3A), peripheral blood (Fig 3B), and bone marrow (Fig 3C) cells from estrogen-treated mice when compared to placebo-treated mice. Consistent with our long-term observation that estrogen decreases spleen cellularity, there was a significant decrease of total splenocytes counts (Fig 3D). However, there was still a significant increase in absolute neutrophil number in the spleen (Fig 3E). Myeloperoxidase (MPO), a major component of azurophilic granules, is most abundantly expressed in neutrophil granulocytes. Accompanying the increased neutrophil counts in the spleen, there was also a substantial upregulation of MPO mRNA (Fig 3F) and protein (Fig 3G) levels in the splenocytes from estrogen-treated B6 mice.

### Estrogen-mediated promotion of inflammatory molecules and neutrophils occurs despite the depletion of NSPs

**Fig 4:**
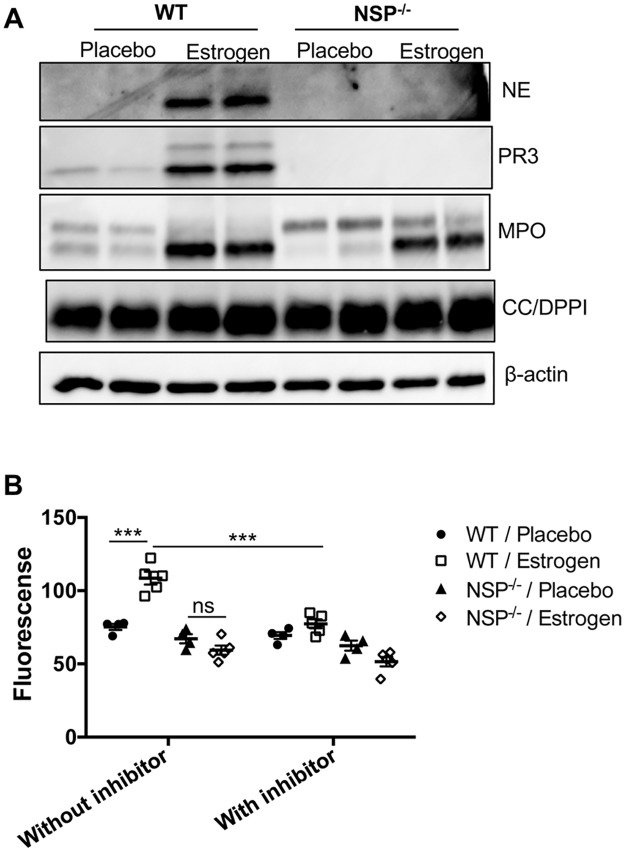
Depletion of NSPs abrogated estrogen-mediated promotion of protease activity in splenocytes. (A) Western blot analysis of NE, PR3, MPO, and CC/DPPI proteins in the splenocytes of placebo and estrogen-treated wild type (WT) and NSP^-/-^ knock out B6 mice. β-actin was probed as protein loading control. (B) Analysis of protease and elastase activity in splenocytes from placebo- and estrogen-treated WT and NSP^-/-^ mice with EnZCheck elastase assay kit. The graph shows the mean ± SEM (n≥4). Unpaired student *t* tests (placebo *vs* estrogen) and paired student *t* test (WT/estrogen; without inhibitor *vs* with inhibitor) were preformed; ***, *p* < 0.001; and ns, not significant.

NSPs have been shown to play an important role in the regulation of inflammatory responses, especially at the site of inflammation [14, 16, 36]. To understand whether increased NSPs contribute to estrogen-mediated promotion of inflammation in splenocytes, we utilized triple NSP knockout (NSP^-/-^) mice and subjected these mice to estrogen treatment. As indicated in Fig 4A, NE and PR3 proteins were not detectable in the splenocytes of NSP^-/-^ mice (in both placebo and estrogen treated mice). Similar to that observed in wild type B6 mice, MPO was also increased in splenocytes from estrogen-treated NSP^-/-^ when compared to placebo-treated NSP^-/-^ mice. CC/DPPI expression level in wild type mice was similar to that in NSP^-/-^ mice, either with or without estrogen treatment (Fig 4A). By using the EnZCheck elastase assay kit, we demonstrated that depletion of NSPs abolished the increase of protease activity in estrogen-treated B6 mice (Fig 4B). This data further suggests that the upregulation of NSPs directly contributed to the observed increase in protease activity in splenocytes from estrogen-treated B6 mice.

**Fig 5:**
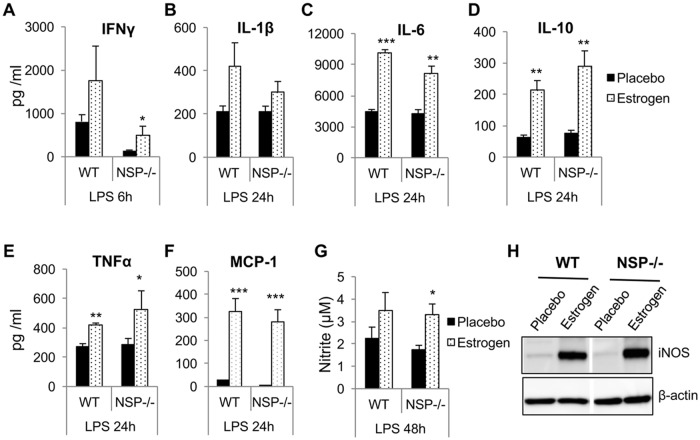
Depletion of NSPs has no obvious effect on estrogen-mediated promotion of inflammatory molecules and neutrophils. The splenocytes from placebo- and estrogen-treated wild type (WT) and NSP^-/-^ knock out mice were stimulated with LPS for the indicated times. The culture supernatant was collected to measure cytokines IFNγ (A), IL-1β (B), IL-6 (C), IL-10 (D), TNFα (E), chemokine MCP-1 (F), and inflammatory molecule NO (G). The expression level of iNOS protein in LPS activated splenocytes (24hr) was measured by Western blotting (H). The graphs show means ± SEMs (n = 3 for wild type B6 mice and n = 5 for NSP^-/-^ mice). Unpaired student *t* test (placebo *vs* estrogen); *, *p* < 0.05; **, *p* < 0.01; and ***, *p* < 0.001.

We initially hypothesized that depletion of NSPs would impede the effect of estrogen on inflammatory mediators in *ex vivo-*activated splenocytes. Nevertheless, similar to estrogen-treated wild type B6 mice, in LPS-activated splenocytes from estrogen-treated NSP^-/-^ triple knock out mice there was enhanced production of inflammatory mediators that include IFNγ (Fig 5A), IL-1β (Fig 5B), IL-6 (Fig 5C), IL-10 (Fig 5D), TNFα (Fig 5E), MCP-1 (Fig 5F), NO (Fig 5G), and iNOS (Fig 5H) when compared to placebo-treated NSP^-/-^ mice.

**Fig 6:**
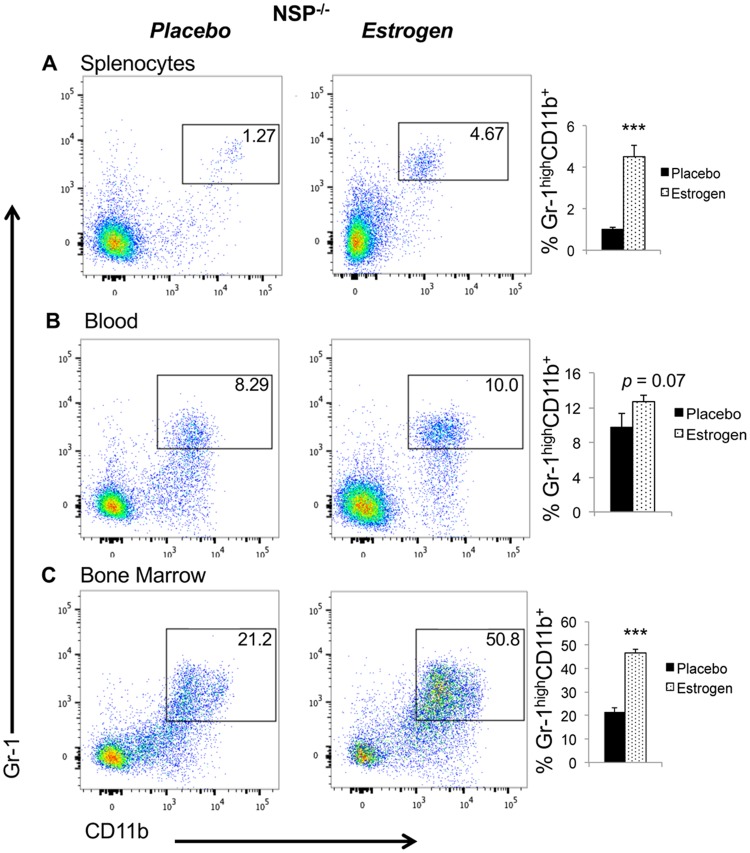
Estrogen treatment increases neutrophil percentages in NSP^-/-^ knock out mice. Red blood cell-depleted whole splenocytes (A), peripheral blood (B), and bone marrow (C) cells from placebo- and estrogen-treated NSP^-/-^ knockout mice were stained with neutrophil surface markers as described in Fig 3. The representative FACS plots are shown. The summary graphs show the percentages of neutrophils (CD11b^+^GR1^+^) in the spleen, blood, and bone marrow cells from placebo- and estrogen-treated NSP^-/-^ mice (means ± SEMs, n≥5). Unpaired student *t* test (placebo *vs* estrogen) were preformed; ***, *p* < 0.001.

In the absence of NSPs, estrogen was still capable of increasing neutrophil percentages in the spleen and bone marrow. Akin to estrogen-treated wild type B6 mice (Fig 3A and 3C), there was also a significant upregulation of Gr-1^high^CD11b^+^ neutrophils in the spleen and bone marrow of estrogen-treated NSP^-/-^ knock out mice when compared to placebo-treated NSP^-/-^ mice (Fig 6A and 6C). The increase of neutrophils in blood was however not significant in estrogen-treated NSP^-/-^ mice (*p* = 0.07, Fig 6B). Consistent with increased neutrophils, the expression of MPO was also increased in estrogen-treated NSP^-/-^ mice when compared with placebo-treated NSP^-/-^ mice (Fig 4A). These data suggest that the effects of estrogen on above inflammatory molecules and neutrophils appear to be independent of estrogen-mediated promotion of NSPs.

### Depletion of splenic neutrophils *in vitro* has limited effect on splenic cell immune responses

**Fig 7:**
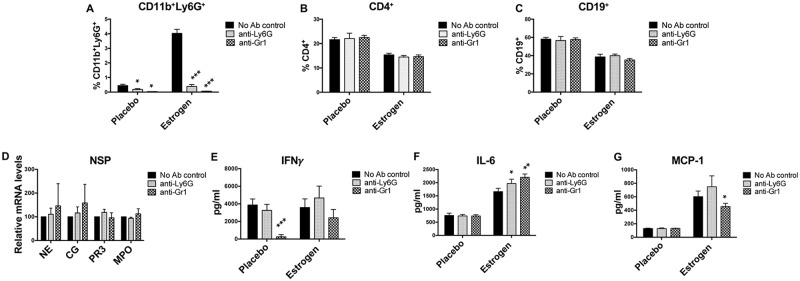
Depletion of neutrophils *in vitro* from splenocytes has limited effect on LPS-induced inflammatory responses in splenocytes. The splenocytes from placebo-and estrogen-treated mice were treated with anti-mouse Ly6G, or anti-mouse Gr-1 antibody and magnetic beads to deplete neutrophils. The No Ab control received no any specific antibody treatment. (A-C) The graphs summarize the flow cytometric analysis data of the percentage of CD11b^+^Ly6G^+^, CD4^+^ and CD19^+^ cells in the splenocytes after neutrophil depletion. (D) Real-time RT-PCR analysis of the relative mRNA expression levels of NE, PR3, and CG in the splenocytes from estrogen-treated B6 mice after neutrophil depletion. The graph represents the means ± SEMs (n = 2). (E-G) The neutrophil-depleted splenocytes from placebo- and estrogen-treated B6 mice were stimulated with LPS for 24h, and then supernatant were collected to analyze the production of IFNγ, IL-6, and MCP-1 by ELISA. The graphs (A-C and E-G) show means ± SEM (n = 2 for placebo with anti-Gr-1 treatment; n≥4 for the other treatment groups). Paired student *t* test (No Ab control *vs* anti-Ly6G or anti-Gr-1); *, *p* < 0.05; **, *p* <0.01; and ***, *p* < 0.001.

The above data indicates that depletion of NSPs has no obvious effect on estrogen-mediated induction of inflammatory cytokines. To further understand the potential biological implication of increased neutrophils in estrogen-treated mice, we depleted splenic neutrophils *in vitro* with either anti-mouse Ly6G (specific for neutrophils) or anti-mouse Gr-1 (neutrophils and monocytes) antibody. As indicated, both anti-mouse Ly6G and Gr-1 efficiently depleted CD11b^+^Ly6G^+^ neutrophils (Fig 7A) from splenocytes, but had no effect on CD4^+^ T (Fig 7B), and CD19^+^ B lymphocytes (Fig 7C). To our surprise, we found that depletion of CD11b^+^Ly6G^+^ neutrophils did not significantly reduce NSP or MPO mRNA expression in the splenocytes (Fig 7D). Also, specific depletion of Ly6G^+^ cells did not reduce LPS-induced IFNγ, IL-6 and MCP-1 (Fig 7E–7G). Indeed, there was a slight, but significant increase of IL-6 in either anti-Ly6G or anti-Gr-1 depleted splenocytes (Fig 7F). However, depletion of Gr-1^+^ cells with anti-Gr-1 antibody suppressed LPS-induced IFNγ in placebo-treated splenocytes and MCP-1 in estrogen-treated splenocytes (Fig 7E and 7G), suggesting a potential involvement of Gr-1^+^ monocytes in LPS-induced IFNγ and MCP-1 in splenocytes. Together, our data suggests that neutrophils have limited effect on LPS-induced inflammatory molecules in splenocytes from placebo- and estrogen-treated mice.

### Increased neutrophils and NSP expression in the spleen cells of different spontaneous murine lupus models

**Fig 8:**
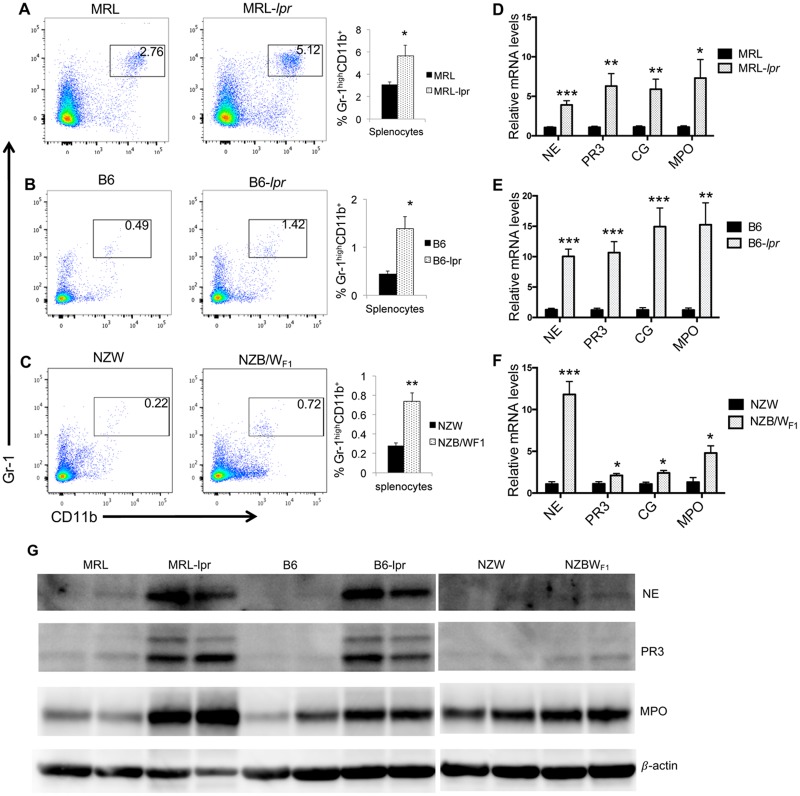
Neutrophil percentages are increased in the splenocytes of three different strains of spontaneous lupus-prone mice. (A-C) The flow cytometric analysis of neutrophils (CD11b^+^GR1^+^) in the splenocytes from MRL-*lpr (A)*, B6-*lpr(B)*, NZB/W_F1_ (C) lupus mice and their respective control MRL, B6, and NZW mice. Representative FACS plots are shown. The graphs show the summary of flow analysis of neutrophil percentage in splenocytes of different strains of lupus mice (means ± SEMs, n ≥4). (D-F) Real-time RT-PCR analysis of the relative expression levels of NE, PR3, CG, and MPO in the splenocytes from MRL-*lpr (D)*, B6-*lpr (E)*, NZB/W_F1_ (F) lupus mice and their respective control MRL, B6, and NZW mice. The graphs show means ± SEM (n ≥4). Unpaired student *t* test (MRL *vs* MRL-*lpr*; and B6 *vs* B6-*lpr*, NZW *vs* NZB/W_F1_); *, *p* < 0.05; **, *p* < 0.01; and ***, *p* < 0.001. (G) Western blot analysis of NE, PR3, and MPO protein expression in splenocytes from MRL-*lpr*, B6-*lpr*, NZBW_F1_, and their respective control MRL, B6, and NZW. β-actin was probed as protein loading control.

Since estrogen is known to regulate autoimmune diseases, we extended our studies to determine whether similar changes to neutrophil numbers and NSPs are also evident in several strains of autoimmune-prone mice. Analogous to our observation in the estrogen-induced murine inflammation model, there was a significant increase in neutrophil percentage in the spleens of autoimmune-prone female MRL-*lpr* (Fig 8A), B6-*lpr* (Fig 8B), and NZB/W_F1_ (Fig 8C) when compared to their respective control female MRL, B6, and NZW mice. However, unlike estrogen-treated B6 mice, there was no increase of neutrophils in the blood and bone marrow of above three strains of autoimmune-prone mice (data not shown). Consistent with increased neutrophil percentage, there were also increased NSP and MPO mRNA expression levels in the splenocytes from MRL-*lpr* (Fig 8D), B6-*lpr* (Fig 8E) and NZB/W_F1_ (Fig 8F) when compared to their respective controls. Western blot analysis indicated that NE, PR3 and MPO protein expression levels were also significantly increased in splenocytes from MRL-*lpr* and B6-*lpr* mice when compared to their respective controls (Fig 8G). While NE and PR3 proteins were increased in NZB/W_F1_ mice (compared to control NZW mice), their expression levels were still very low. Since MPO protein was already expressed at relatively high levels in the NZW control, the increase of MPO protein levels in NZB/W_F1_ was not as prominent as that in *lpr* lupus mice (Fig 8G). Together, our data indicates an increase of neutrophil percentage and NSP expression in the spleens of different spontaneous lupus strains.

## Discussion

There is now a wealth of data indicating that estrogen regulates the development and functions of T cell subsets (Th1, Th2, Treg) and B cell subsets (B1 and B2) [1, 11, 13, 37, 38]. Estrogen is also known to regulate the development, differentiation and functions of dendritic cells, macrophages and monocytes [1, 39]. However, the data with regards to the regulatory effect of estrogen on neutrophils are limited.

Neutrophils have a short life span and therefore are constantly replenished from bone marrow. The sex differences in neutrophil numbers suggest a potential regulatory role of sex hormones such as estrogen. Studies in humans have shown neutrophilia in pregnant women correlated with increased levels of progesterone and estrogen throughout pregnancy[23, 40]. Moreover, increased neutrophil numbers were also seen in cancer patients who received estramustine phosphate chemotherapy treatment, which elevates serum 17-β estradiol level [41]. Nevertheless, in one older murine study, estrogen appeared to have suppressive effects on hematopoiesis and granulopoiesis, since administration of estrogen blocked the increase of circulating lymphocytes, neutrophils, and monocytes in ovariectomized mice [42]. Together, these data suggest a regulatory effect of estrogen on neutrophils. Here, we report an increase in neutrophil percentages in the primary lymphoid organ bone marrow, peripheral blood and in the secondary lymphoid organ spleen in *in vivo* estrogen-treated B6 mice (Fig 3). Despite a reduction of total splenic cellularity, the absolute splenic neutrophil counts were increased (Fig 3E), in addition to increased percentage (Fig 3A). Since 7–8 wks of estrogen implant treatment in mice causes osteopetrosis and diminishes the bone marrow cavity, we were unable to recover all the bone marrow cells from estrogen-treated mice to ascertain the absolute number of bone marrow neutrophils. The precise reasons for estrogen-mediated increase in splenic neutrophils remains unclear. It is plausible that estrogen treatment stimulates splenic lymphoid cells to secrete neutrophil attracting chemokines or promotes localized development and/or survival of neutrophils. This hypothesis is supported by the reports that estrogen promotes the production of neutrophil-attracting chemokine MCP-1 in splenocytes [8] and that estrogen delays spontaneous neutrophil apoptosis to induce neutrophilia in pregnant women [40]. Further, although the mice appeared healthy after estrogen treatment, we cannot rule out the possibility that estrogen may causes an undetermined endogenous infection that enhanced neutrophil numbers and activities. We have initially utilized anti-CD11b and anti-GR-1 antibodies to identify neutrophils in this study. Since Gr-1 also labels Ly6C on eosinophils and monocytes, we later used anti-CD11b and a specific anti-Ly6G antibody to confirm the increase of neutrophils (CD11b^+^Ly6G^+^) in the spleens of estrogen-treated B6 mice (S1 Fig).

In agreement with the increase in neutrophil counts in splenocytes of estrogen-treated mice, we observed an increase in NSPs and neutrophil specific MPO expression (Figs 2 and 3). The increase in NSP expression is not simply due to increased neutrophil counts in the spleen since depletion of neutrophils *in vitro* from splenocytes did not reduce NSP expression levels. We also analyzed NSP expression in both purified CD11b^+^ myeloid cells and CD90.2^+^ T cells from placebo- and estrogen-treated B6 mice. We found that estrogen-treatment increased NSPs and MPO mRNA expression not only in CD11b^+^ myeloid cells, but also in CD90.2^+^ T cells (S2A–S2D Fig). The increase of NE and MPO mRNA expression was also observed in purified splenic CD4^+^ T and CD19^+^ B cells from MRL-*lpr* mice when compared to that from control MRL mice (S2E and S2F Fig). Further investigation is necessary to determine the functionality of the NSPs in T cells.

Both estrogen receptor (ER)α and ERβ are expressed in neutrophils and regulate neutrophil functions [23]. It has been reported that ERα plays an essential role in estrogen-mediated regulation of inflammatory responses in different immune cell types [9, 43]. Due to an unexpected loss of ERα^-/-^ mice during the estrogen treatment, we can only draw limited conclusions from the reduced number of surviving estrogen-treated ERα^-/-^ knock out mice study. Consistent with previous reports, our limited data indicate that depletion of ERα abolished the positive effect of estrogen on the induction of inflammatory molecules such as NO (S3A Fig), iNOS (S3B Fig), and MCP-1 (S3C Fig) in activated splenocytes. Moreover, depletion of ERα eliminated estrogen-mediated upregulation of NSPs in the spleen (S3D Fig). This data is suggestive of the involvement of ERα in estrogen-mediated promotion of inflammation and NSPs in splenocytes, with the caveat that the limited number of estrogen-treated ERα^-/-^ mice preclude drawing firm conclusions.

Previous studies have shown that the absence of NSPs led to defective onsite cytokine production *in vivo* in various models of inflammation [36, 44]. However, NSP deficient neutrophils showed no defect in cytokine/chemokine production when directly stimulated *in vitro* with PMA or LPS [45]. Similarly, we noted that there were no differences in the production of inflammatory mediators (IFNγ, IL-1β, IL-6, TNFα, MCP-1 and iNOS/NO) between LPS-activated wild type B6 and NSP^-/-^ splenocytes (Fig 5). Estrogen treatment promoted inflammatory responses in activated NSP^-/-^ splenocytes similar to that noted in wild type B6 mice splenocytes (Fig 5). These data suggest that the *ex vivo* promotion of aforementioned inflammatory molecules by estrogen in splenocytes might be mediated through NSP-independent mechanisms.

Depletion of neutrophils specifically with anti-mouse Ly6G antibody *in vitro* from splenocytes did not have an obvious effect on LPS-induced immune responses in splenocytes, while depletion of both neutrophils and Gr-1^+^ monocytes with anti-mouse Gr-1 antibody had a suppressive effect on LPS-induced IFNγ and MCP-1. Remarkably, depletion of neutrophils also did not affect the expression of NSP mRNAs suggesting that other cells may contribute to NSP synthesis. In support of this view, we noticed that estrogen promoted NSPs in T lymphocytes (S2 Fig).

Although the direct functional relevance of increased neutrophil counts and NSP expression in splenic cells in estrogen-mediated promotion of inflammation remains unknown, the similar upregulation of splenic neutrophils and NSPs in estrogen-treated B6 mice and in spontaneous autoimmune-prone murine models implicates a potential significance of neutrophils in estrogen mediated autoinflammatory responses. Together, our studies provide new information about the role of estrogen in neutrophils and NSPs, which bear remarkable similarity to that noticed in several autoimmune prone female mice. The precise contribution of altered neutrophils and NSPs to the activity of other defined splenic cell subsets warrants further investigation in future studies, which shall provide new perspective for understanding the cellular mechanism of estrogen regulation of inflammation and autoimmunity.

## Supporting information

S1 Fig Increase of CD11b^+^Ly6G^+^ neutrophils in estrogen-treated wild type B6 mice. Red blood cell-depleted whole splenocytes placebo-and estrogen-treated mice were stained with neutrophil surface markers PE conjugated anti-Ly6G and PerCP-Cy5.5 conjugated anti-CD11b antibodies. The representative flow cytometry plots are shown. The bar graph shows the mean ± SEMs percentages of CD11b^+^Ly6G^+^ neutrophils in the splenocytes from placebo- and estrogen-treated mice (n≥4).
(TIF)

S2 Fig NSPs and MPO mRNA expression levels are increased in lymphocytes from estrogen-treated B6 mice and MRL-*lpr* mice. Splenic CD11b^+^ myeloid lineage cells, CD90.2^+^ T, CD4^+^ T and CD19^+^ B cells were purified by positive selection, per manufacturer’s instruction, using mouse CD11b and CD90.2 (Thy1.2), CD4 (L3T4), and CD19 microbeads (Miltenyi Biotec, San Diego, CA, USA), **(A-D)**. Real-time RT-PCR analysis of the relative mRNA expression levels of NE (A), PR3 (B), CG (C), and MPO (D) in purified splenic CD11b^+^ and CD90.2^+^ cells from placebo- and estrogen-treated B6 mice. The graphs represent means ± SEMs (n = 4 each). (E and F) Real-time RT-PCR analysis of the relative mRNA expression levels of NE (E) and MPO (F) in purified splenic CD4^+^ T and CD19^+^ B cells from MRL-*lpr* and control MRL mice. The graphs represent means ± SEMs (n = 2 each).
(TIF)

S3 Fig Depletion of ERα abolished the promotion effect of estrogen on inflammatory responses and NSPs. The 4–5 wks old, male ER knock out mice (ER^-/-^, purchased from the Jackson laboratory, USA) were orchidectomized and implanted with empty (placebo control) or 17-β estradiol silastic implants as we described for wild type B6 mice in the material and method section. The splenocytes from placebo- and estrogen-treated wild type (WT) and ERα^-/-^ knock out mice were stimulated with Con A or LPS for either 24hrs or 48hrs to measure the production of inflammatory molecules such as NO (A) and MCP-1 (C) in culture supernatant. Western blotting was performed to detect iNOS protein expression in Con A activated splenocytes (24hr) (B). (D) Real-time RT-PCR analysis of NSP expression in freshly isolated splenocytes. The graph shows means ± SEM (n = 1 for estrogen-treated ERα^-/-^; n = 2 for the other treatment groups).
(TIF)

## References

[1] Lang TJ. Estrogen as an immunomodulator. Clinical immunology (Orlando, Fla. 2004;113(3):224–30. 10.1016/j.clim.2004.05.011 15507385

[2] Ahmed SA, Hissong BD, Verthelyi D, Donner K, Becker K, Karpuzoglu-Sahin E. Gender and risk of autoimmune diseases: possible role of estrogenic compounds. Environ Health Perspect. 1999;107 Suppl 5:681–6. 10.1289/ehp.99107s5681 PMC1566250 10502531

[3] Ansar Ahmed S, Penhale WJ, Talal N. Sex hormones, immune responses, and autoimmune diseases. Mechanisms of sex hormone action. The American journal of pathology. 1985;121(3):531–51. 3907369 PMC1887926

[4] Cutolo M, Capellino S, Sulli A, Serioli B, Secchi ME, Villaggio B, et al Estrogens and autoimmune diseases. Annals of the New York Academy of Sciences. 2006;1089:538–47. 10.1196/annals.1386.043 17261796

[5] Khan D, Cawan C, Ahmed SA. Estrogen and Signaling in the Cells of Immune System. Advances in Neuroimmune Biology. 2012;3(1):73–93. Epub 2012/05/29.

[6] Karpuzoglu-Sahin E, Hissong BD, Ansar Ahmed S. Interferon-gamma levels are upregulated by 17-beta-estradiol and diethylstilbestrol. J Reprod Immunol. 2001;52(1–2):113–27. 11600182 10.1016/s0165-0378(01)00117-6

[7] Calippe B, Douin-Echinard V, Laffargue M, Laurell H, Rana-Poussine V, Pipy B, et al Chronic estradiol administration in vivo promotes the proinflammatory response of macrophages to TLR4 activation: involvement of the phosphatidylinositol 3-kinase pathway. J Immunol. 2008;180(12):7980–8. 18523261 10.4049/jimmunol.180.12.7980

[8] Lengi AJ, Phillips RA, Karpuzoglu E, Ahmed SA. Estrogen selectively regulates chemokines in murine splenocytes. J Leukoc Biol. 2007;81(4):1065–74. 10.1189/jlb.0606391 17185357

[9] Calippe B, Douin-Echinard V, Delpy L, Laffargue M, Lelu K, Krust A, et al 17Beta-estradiol promotes TLR4-triggered proinflammatory mediator production through direct estrogen receptor alpha signaling in macrophages in vivo. J Immunol. 2010;185(2):1169–76. 10.4049/jimmunol.0902383 20554954

[10] Verthelyi DI, Ahmed SA. Estrogen increases the number of plasma cells and enhances their autoantibody production in nonautoimmune C57BL/6 mice. Cell Immunol. 1998;189(2):125–34. 10.1006/cimm.1998.1372 9790726

[11] Ansar Ahmed S, Dauphinee MJ, Montoya AI, Talal N. Estrogen induces normal murine CD5+ B cells to produce autoantibodies. J Immunol. 1989;142(8):2647–53. 2467934

[12] Bynoe MS, Grimaldi CM, Diamond B. Estrogen up-regulates Bcl-2 and blocks tolerance induction of naive B cells. Proceedings of the National Academy of Sciences of the United States of America. 2000;97(6):2703–8. 10.1073/pnas.040577497 10694576 PMC15993

[13] Grimaldi CM, Cleary J, Dagtas AS, Moussai D, Diamond B. Estrogen alters thresholds for B cell apoptosis and activation. J Clin Invest. 2002;109(12):1625–33. 10.1172/JCI14873 12070310 PMC151010

[14] Pham CT. Neutrophil serine proteases: specific regulators of inflammation. Nature reviews. 2006;6(7):541–50. Epub 2006/06/27. 10.1038/nri1841 16799473

[15] Amulic B, Cazalet C, Hayes GL, Metzler KD, Zychlinsky A. Neutrophil function: from mechanisms to disease. Annu Rev Immunol. 2012;30:459–89. Epub 2012/01/10. 10.1146/annurev-immunol-020711-074942 22224774

[16] Wiedow O, Meyer-Hoffert U. Neutrophil serine proteases: potential key regulators of cell signalling during inflammation. J Intern Med. 2005;257(4):319–28. Epub 2005/03/25. 10.1111/j.1365-2796.2005.01476.x 15788001

[17] Pham CT. Neutrophil serine proteases fine-tune the inflammatory response. Int J Biochem Cell Biol. 2008;40(6–7):1317–33. Epub 2008/01/09. 10.1016/j.biocel.2007.11.008 18180196 PMC2440796

[18] Shpacovitch V, Feld M, Hollenberg MD, Luger TA, Steinhoff M. Role of protease-activated receptors in inflammatory responses, innate and adaptive immunity. J Leukoc Biol. 2008;83(6):1309–22. Epub 2008/03/19. 10.1189/jlb.0108001 18347074

[19] Muller I, Munder M, Kropf P, Hansch GM. Polymorphonuclear neutrophils and T lymphocytes: strange bedfellows or brothers in arms? Trends Immunol. 2009;30(11):522–30. 10.1016/j.it.2009.07.007 19775938

[20] Tillack K, Breiden P, Martin R, Sospedra M. T lymphocyte priming by neutrophil extracellular traps links innate and adaptive immune responses. J Immunol. 2012;188(7):3150–9. Epub 2012/02/22. 10.4049/jimmunol.1103414 22351936

[21] Bain BJ, England JM. Normal haematological values: sex difference in neutrophil count. Br Med J. 1975;1(5953):306–9. 1111792 10.1136/bmj.1.5953.306 PMC1672487

[22] Bain BJ, England JM. Variations in leucocyte count during menstrual cycle. Br Med J. 1975;2(5969):473–5. 1148661 10.1136/bmj.2.5969.473 PMC1673417

[23] Blesson CS. Estrogen Receptors in Leukocytes—Possible Impact on Inflammatory Processes in the Female Reproductive System. In: Aimaretti G, editor. Update on Mechanisms of Hormone Action—Focus on Metabolism, Growth and Reproduction: InTech; 2011. http://www.intechopen.com/books/update-on-mechanisms-of-hormone-action-focus-on-metabolism-growth-and-reproduction/estrogen-receptors-in-leukocytes-possible-impact-on-inflammatory-processes-in-the-female-reproductiv

[24] Mathur S, Mathur RS, Goust JM, Williamson HO, Fudenberg HH. Cyclic variations in white cell subpopulations in the human menstrual cycle: correlations with progesterone and estradiol. Clin Immunol Immunopathol. 1979;13(3):246–53. 313307 10.1016/0090-1229(79)90069-2

[25] Crandall TL, Joyce RA, Boggs DR. Estrogens and hematopoiesis: characterization and studies on the mechanism of neutropenia. The Journal of laboratory and clinical medicine. 1980;95(6):857–67. 6966670

[26] Gaunt SD, Pierce KR. Myelopoiesis and marrow adherent cells in estradiol-treated mice. Veterinary pathology. 1985;22(4):403–8. 4035944 10.1177/030098588502200416

[27] Dietsch V, Kalf GF, Hazel BA. Induction of granulocytic differentiation in myeloblasts by 17-beta-estradiol involves the leukotriene D4 receptor. Receptors & signal transduction. 1996;6(2):63–75. 9015862

[28] Miller AP, Feng W, Xing D, Weathington NM, Blalock JE, Chen YF, et al Estrogen modulates inflammatory mediator expression and neutrophil chemotaxis in injured arteries. Circulation. 2004;110(12):1664–9. 10.1161/01.CIR.0000142050.19488.C7 15353495

[29] Robinson DP, Hall OJ, Nilles TL, Bream JH, Klein SL. 17beta-estradiol protects females against influenza by recruiting neutrophils and increasing virus-specific CD8 T cell responses in the lungs. J Virol. 2014;88(9):4711–20. 10.1128/JVI.02081-13 24522912 PMC3993800

[30] Korkmaz B, Horwitz MS, Jenne DE, Gauthier F. Neutrophil elastase, proteinase 3, and cathepsin G as therapeutic targets in human diseases. Pharmacol Rev. 2010;62(4):726–59. Epub 2010/11/17. 10.1124/pr.110.002733 21079042 PMC2993259

[31] Yan H, Zhou HF, Akk A, Hu Y, Springer LE, Ennis TL, et al Neutrophil Proteases Promote Experimental Abdominal Aortic Aneurysm via Extracellular Trap Release and Plasmacytoid Dendritic Cell Activation. Arterioscler Thromb Vasc Biol. 2016;36(8):1660–9. 10.1161/ATVBAHA.116.307786 27283739 PMC4965335

[32] Karpuzoglu E, Fenaux JB, Phillips RA, Lengi AJ, Elvinger F, Ansar Ahmed S. Estrogen up-regulates inducible nitric oxide synthase, nitric oxide, and cyclooxygenase-2 in splenocytes activated with T cell stimulants: role of interferon-gamma. Endocrinology. 2006;147(2):662–71. 10.1210/en.2005-0829 16293660

[33] Dai R, Phillips RA, Ahmed SA. Despite inhibition of nuclear localization of NF-kappa B p65, c-Rel, and RelB, 17-beta estradiol up-regulates NF-kappa B signaling in mouse splenocytes: the potential role of Bcl-3. J Immunol. 2007;179(3):1776–83. 17641044 10.4049/jimmunol.179.3.1776

[34] Dai R, Phillips RA, Zhang Y, Khan D, Crasta O, Ahmed SA. Suppression of LPS-induced Interferon-gamma and nitric oxide in splenic lymphocytes by select estrogen-regulated microRNAs: a novel mechanism of immune modulation. Blood. 2008;112(12):4591–7. 10.1182/blood-2008-04-152488 18791161 PMC2597130

[35] Dai R, Phillips RA, Karpuzoglu E, Khan D, Ahmed SA. Estrogen regulates transcription factors STAT-1 and NF-kappaB to promote inducible nitric oxide synthase and inflammatory responses. J Immunol. 2009;183(11):6998–7005. 10.4049/jimmunol.0901737 19890039 PMC2782783

[36] Adkison AM, Raptis SZ, Kelley DG, Pham CT. Dipeptidyl peptidase I activates neutrophil-derived serine proteases and regulates the development of acute experimental arthritis. J Clin Invest. 2002;109(3):363–71. Epub 2002/02/06. 10.1172/JCI13462 11827996 PMC150852

[37] Salem ML. Estrogen, A Double-Edged Sword: Modulation of TH1- and TH2-Mediated Inflammations by Differential Regulation of TH1/TH2 Cytokine Production. Current drug targets. 2004;3(1):97–104. 15032646 10.2174/1568010043483944

[38] Grimaldi CM, Hill L, Xu X, Peeva E, Diamond B. Hormonal modulation of B cell development and repertoire selection. Mol Immunol. 2005;42(7):811–20. 10.1016/j.molimm.2004.05.014 15829269

[39] Kovats S. Estrogen receptors regulate an inflammatory pathway of dendritic cell differentiation: mechanisms and implications for immunity. Hormones and behavior. 2012;62(3):254–62. 10.1016/j.yhbeh.2012.04.011 22561458 PMC3415586

[40] Molloy EJ, O'Neill AJ, Grantham JJ, Sheridan-Pereira M, Fitzpatrick JM, Webb DW, et al Sex-specific alterations in neutrophil apoptosis: the role of estradiol and progesterone. Blood. 2003;102(7):2653–9. 10.1182/blood-2003-02-0649 12791649

[41] Suzuki M, Fujimura T, Fukuhara H, Enomoto Y, Nishimatsu H, Ishikawa A, et al 17β-Estradiol-Mediated Elevation of Peripheral White Blood Cell Count During Estramustine Phosphate Therapy for Prostate Cancer. Int J Endocrinol Metab. 2011;9(4):347–51.

[42] Jilka RL, Passeri G, Girasole G, Cooper S, Abrams J, Broxmeyer H, et al Estrogen loss upregulates hematopoiesis in the mouse: a mediating role of IL-6. Exp Hematol. 1995;23(6):500–6. 7768305

[43] Lelu K, Laffont S, Delpy L, Paulet PE, Perinat T, Tschanz SA, et al Estrogen receptor alpha signaling in T lymphocytes is required for estradiol-mediated inhibition of Th1 and Th17 cell differentiation and protection against experimental autoimmune encephalomyelitis. J Immunol. 2011;187(5):2386–93. 10.4049/jimmunol.1101578 21810607

[44] Schreiber A, Pham CT, Hu Y, Schneider W, Luft FC, Kettritz R. Neutrophil serine proteases promote IL-1beta generation and injury in necrotizing crescentic glomerulonephritis. J Am Soc Nephrol. 2012;23(3):470–82. 10.1681/ASN.2010080892 22241891 PMC3294298

[45] Raptis SZ, Shapiro SD, Simmons PM, Cheng AM, Pham CT. Serine protease cathepsin G regulates adhesion-dependent neutrophil effector functions by modulating integrin clustering. Immunity. 2005;22(6):679–91. 10.1016/j.immuni.2005.03.015 15963783

