# Supplementary figures and images for: Neutrophils and neutrophil serine proteases are increased in the spleens of estrogen-treated C57BL/6 mice and several strains of spontaneous lupus-prone mice
Source: PLoS One. 2017 Feb 13;12(2):e0172105. doi: 10.1371/journal.pone.0172105 (PMC5305105; doi:10.1371/journal.pone.0172105)

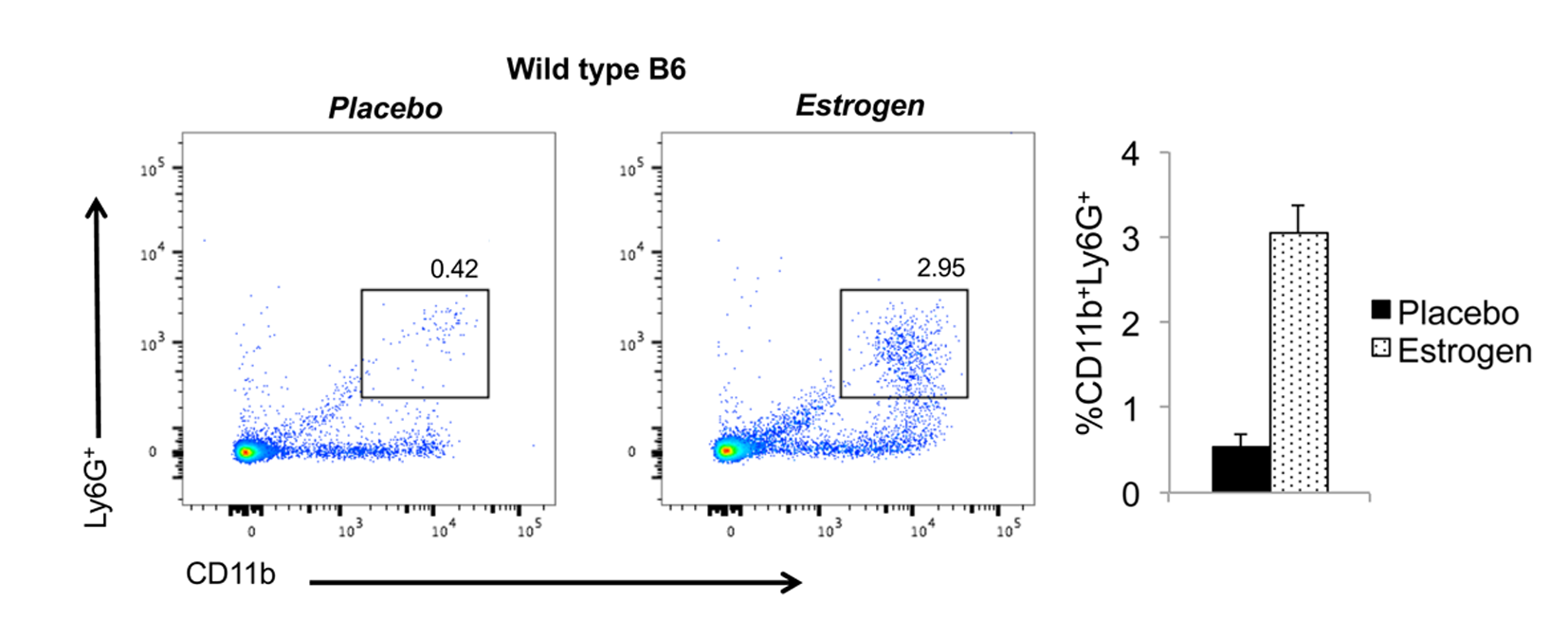

Supplement: S1 Fig — Red blood cell-depleted whole splenocytes placebo-and estrogen-treated mice were stained with neutrophil surface markers PE conjugated anti-Ly6G and PerCP-Cy5.5 conjugated anti-CD11b antibodies. The representative flow cytometry plots are shown. The bar graph shows the mean ± SEMs percentages of CD11b+Ly6G+ neutrophils in the splenocytes from placebo- and estrogen-treated mice (n≥4). (TIF) [file pone.0172105.s001.tif]

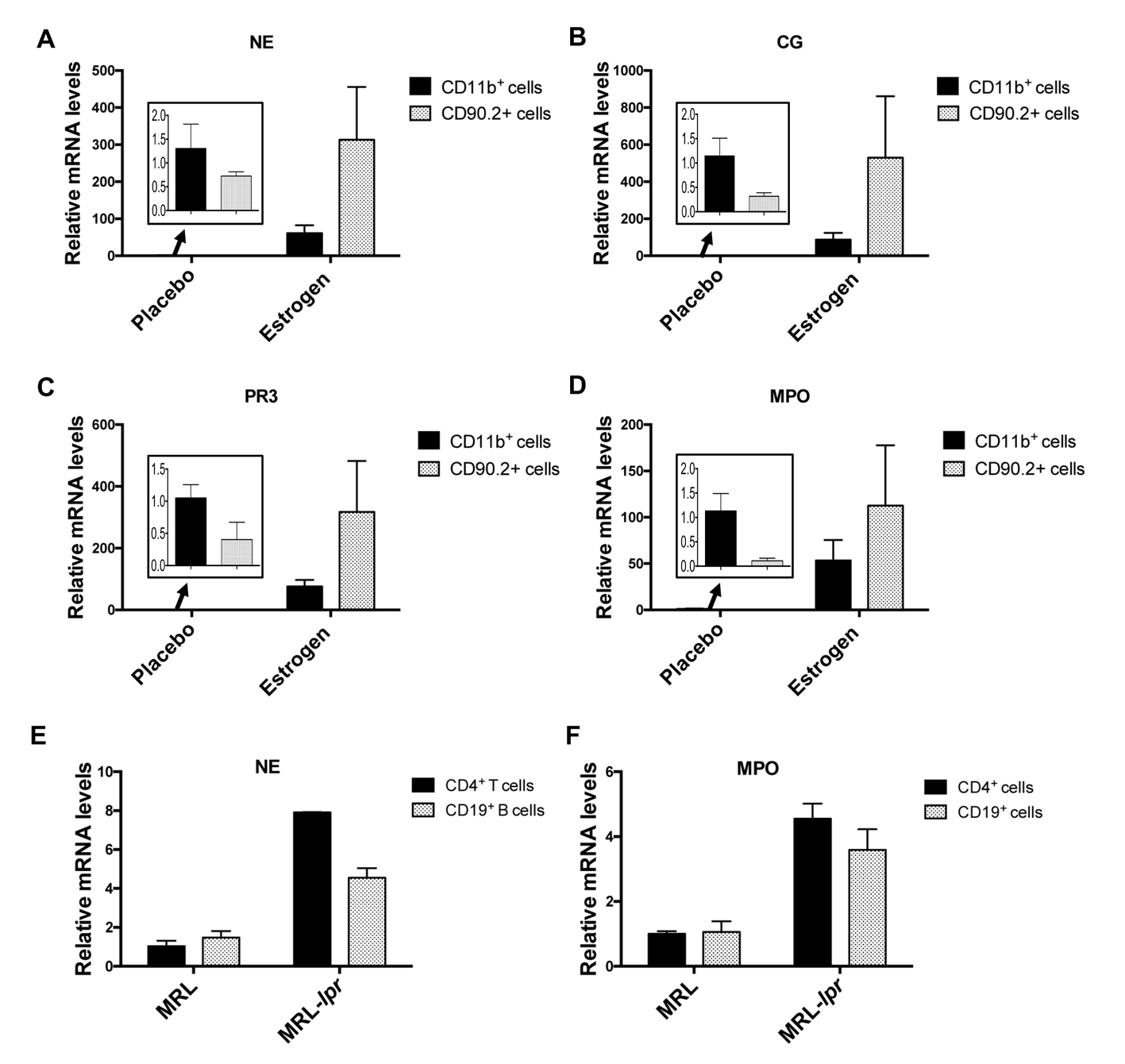

Supplement: S2 Fig — Splenic CD11b+ myeloid lineage cells, CD90.2+ T, CD4+ T and CD19+ B cells were purified by positive selection, per manufacturer’s instruction, using mouse CD11b and CD90.2 (Thy1.2), CD4 (L3T4), and CD19 microbeads (Miltenyi Biotec, San Diego, CA, USA), (A-D). Real-time RT-PCR analysis of the relative mRNA expression levels of NE (A), PR3 (B), CG (C), and MPO (D) in purified splenic CD11b+ and CD90.2+ cells from placebo- and estrogen-treated B6 mice. The graphs represent means ± SEMs (n = 4 each). (E and F) Real-time RT-PCR analysis of the relative mRNA expression levels of NE (E) and MPO (F) in purified splenic CD4+ T and CD19+ B cells from MRL-lpr and control MRL mice. The graphs represent means ± SEMs (n = 2 each). (TIF) [file pone.0172105.s002.tif]

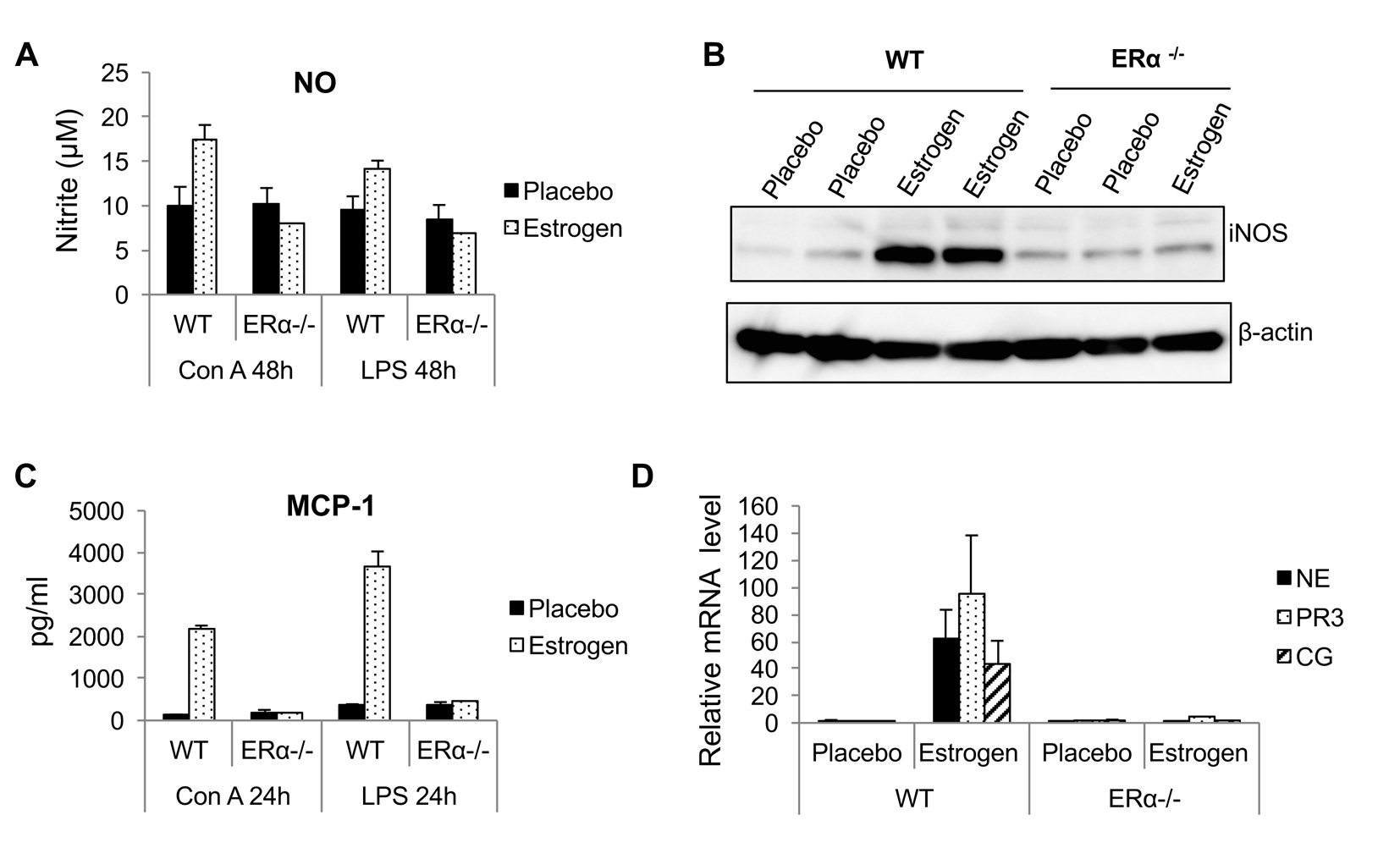

Supplement: S3 Fig — The 4–5 wks old, male ER knock out mice (ER-/-, purchased from the Jackson laboratory, USA) were orchidectomized and implanted with empty (placebo control) or 17-β estradiol silastic implants as we described for wild type B6 mice in the material and method section. The splenocytes from placebo- and estrogen-treated wild type (WT) and ERα-/- knock out mice were stimulated with Con A or LPS for either 24hrs or 48hrs to measure the production of inflammatory molecules such as NO (A) and MCP-1 (C) in culture supernatant. Western blotting was performed to detect iNOS protein expression in Con A activated splenocytes (24hr) (B). (D) Real-time RT-PCR analysis of NSP expression in freshly isolated splenocytes. The graph shows means ± SEM (n = 1 for estrogen-treated ERα-/-; n = 2 for the other treatment groups). (TIF) [file pone.0172105.s003.tif]
